# Jail Healthcare Staffing in the US Southeast: a Cross-Sectional Survey

**DOI:** 10.1007/s11606-023-08454-3

**Published:** 2023-10-26

**Authors:** David L. Rosen, Jessica Carda-Auten, Elena DiRosa, Debbie Travers

**Affiliations:** 1 Institute for Global Health and Infectious Diseases, University of North Carolina at Chapel Hill, Chapel Hill, NC USA; 2 School of Nursing, University of North Carolina at Chapel Hill, Chapel Hill, NC USA; 3 Present Address: School of Nursing, Duke University, Durham, NC USA

## Abstract

**Background:**

Jails annually incarcerate millions of people with health problems, yet jail healthcare services have not been well described.

**Objective:**

To describe jail healthcare staffing.

**Design:**

Phone-administered survey conducted October 2020 to May 2021.

**Setting:**

County jails in North Carolina, South Carolina, Georgia, and Alabama.

**Participants:**

Jail personnel “most knowledgeable” about jail healthcare.

**Main Measures:**

Weekly on-site healthcare coverage rate (hours per 100 incarcerated person-weeks [IPWs]) by personnel type; telemedicine rates and detention officers’ healthcare duties.

**Key Results:**

Survey response rate was 73% (254/346). Among surveyed jails, 71% had on-site non-psychiatric providers (e.g., physicians, physician assistants) (median of 3.3 h per 100 IPWs); 90% had on-site nursing (median of 57.0 h per 100 IPWs) including 50% with on-site registered nurses (median of 25 h per 100 IPWs) and 70% with on-site licensed practical nurses (median of 52 h per 100 IPWs); 9% had on-site psychiatric providers (median of 1.6 h per 100 PWs). Telemedicine was used for primary care in 13% of jails (median 2.1 h per 100 IPW); for mental healthcare in 55% (median 2.1 h per 100 IPW); and for other specialties in 5% (median 1.0 h per 100 IPW). In 81% of jails, officers conducted medical intake and in 58% assessed urgency of medical requests (i.e., “sick call”). The number of officers’ healthcare responsibilities increased inversely with weekly nursing coverage.

**Conclusions:**

Nearly 30% of surveyed jails routinely lacked on-site healthcare providers and in most other jails providers’ on-site presence was modest. Jails relied heavily on LPNs and officers for care, resulting in missed opportunities for care and potentially endangering incarcerated persons.

**Supplementary Information:**

The online version contains supplementary material available at 10.1007/s11606-023-08454-3.

## Introduction

In 2020, there were nearly 9 million US jail admissions.^1^ Black and Native people and financially impoverished people are jailed at disproportionately high rates.^1, 2^ The burden of health problems among jailed people is substantial. In a 2011–2012 national survey of jailed people, 40% reported a current chronic medical condition^3^ and 44% reported a history of mental disorders.^4^ In an earlier nationwide study, 63% of people sentenced to jail met the criteria for substance abuse or dependence, an estimate nearly 15 times that of the general population.^5^

The need for adequate jail healthcare is a long-standing concern. A 1972 American Medical Association survey raised awareness about the insufficiencies of jail healthcare,^6, 7^ finding that fewer than 40% had available physicians and 66% had “first aid facilities” only.^8^ In 1976, the US Supreme Court’s ruling in *Estelle v. Gamble* established that jails and prisons have an obligation to provide healthcare.^9^ But the legal standard for care, which prohibits willfully ignoring “serious medical needs,” is ill-defined,^10^ and unlike other healthcare settings, jails and prisons have little regulatory oversight.

Some healthcare challenges among jails are distinct. Unlike prisons, which are operated within the federal or state *systems*, jails are typically operated by individual counties under the sheriff’s supervision; jails have relatively modest resources and can achieve fewer healthcare economies of scale than those of prison systems. Unlike prisons, jails predominantly incarcerate people awaiting trial or sentenced to one year or less.^11, 12^ As a result, the numbers of entries and exits within US jails are more than 25 times those of prisons. Although typical jail incarceration is 2 or 3 days, 25% are 1 week or longer and a small proportion are months or years.^13–15^ Consequently, jails must provide care for large numbers of people with—sometimes emergent—health issues, with many people released within a few days, but some requiring chronic care over longer periods.

Data from the 523 largest US jails suggest that healthcare contracting is increasingly common; from 2009 to 2018, healthcare contracting among these jails increased from 48 to 63%.^16^ The use of contracting in smaller jails, which comprise the majority of the ~3200 US jails, is unknown.^17^ Nevertheless, employing contractors, particularly large for-profit correctional healthcare companies, has raised concerns that these businesses prioritize profits at the expense of providing adequate care.^16^

In the past 50 years, there has been little effort to characterize jail healthcare staffing. In a qualitative study of 34 jails in the US southeast, healthcare staffing varied greatly across jails and detention officers had a host of healthcare duties.^18^ In the current study, we sought to quantitatively characterize jail healthcare staffing among jails in the southeast, a region with comparatively high rates of mortality,^19, 20^ un-insurance,^21^ and incarceration.^17^

## Methods

### Study Population and Recruitment

From October 2020 to May 2021, we conducted a phone survey to assess healthcare services available in county jails located within four southeastern states: North Carolina (NC), South Carolina (SC), Georgia (GA), and Alabama (AL). We identified the universe of county jails in these states using the Bureau of Justice Statistics Census and Surveys of jails, and sheriff association directories, and by searching the Internet. “City jails” which often serve as short-term (i.e., a few hours) “holding tanks” were excluded. We contacted the sheriff, jail administrator, or jail healthcare administrator to identify the person at each jail recognized as the “most knowledgeable” about jail healthcare resources, staffing, and practices. Among jails with on-site healthcare personnel, those personnel were typically identified as most knowledgeable. When a jail had no on-site healthcare staffing or when healthcare personnel could not be reached, declined to participate, or were barred from participating by their contracted company, we attempted to survey jail administrators. Surveys were conducted by phone, requiring about 45 min to complete. Participants were offered $35 for participation.

### Survey Development and Domains

Survey development was informed by a literature review, qualitative findings,^18^ expert input, and cognitive testing and piloting (see Appendix 1 for more details). The survey (Appendix 2) included the following domains relevant to this analysis: (1) jail healthcare personnel; (2) weekly healthcare on-site coverage (i.e., days of week, hours of the day) by personnel type; (4) telemedicine use; and (5) detention officer healthcare duties. We also collected information to characterize study jails (e.g., daily population, healthcare contracting) and our respondents (e.g., demographics), and we used public data to compare the characteristics of surveyed jails (i.e., population size,^22^ rurality of surrounding county^23^) with non-response jails.

### Analysis

We tested for differences between jails for which we did and did not have responses, comparing their distributions of characteristics using chi-square tests.

We grouped healthcare personnel into five types: non-psychiatric providers, which we refer to as *providers* (i.e., Doctors of Medicine [MD], Doctors of Osteopathic Medicine [DO], Physician Assistants [PA], and Nurse Practitioners [NP]), *nurses* (i.e., Registered Nurses [RN], Licensed Practicing Nurses [LPN], and Paramedics), *nursing assistants* (i.e., Certified Nursing Assistant, Certified Medical Assistant, and Medical Technician), *psychiatric providers* (i.e., MD, DO, PA, or NP), and *other mental health personnel* (e.g., psychologist, clinical social worker, or counselor).

For each healthcare personnel type, we estimated the percentage of jails with any staffing, regardless if contracted or not, and including those providing services on-site, on-call, or via telemedicine. Then, for each healthcare personnel type, we estimated “weekly coverage rates,” defined as the total *on-site* hours in a week—with a possible maximum of 168 h—divided by the jails’ population, which was assessed on the day of the survey and assumed to be stable throughout a typical week. We multiplied rates by 100 to report weekly hours of coverage per 100 incarcerated person-weeks (IPW). We then generated a second set of estimates, recalculating rates, but only including jails that staffed the corresponding personnel type (e.g., jails with no on-site RNs were not included in the estimate of median RN coverage hours). We refer to these two sets of estimates as those for “All study jails” and those for “Staffed study jails.” For both sets of rates, we described their distribution by reporting their medians and interquartile ranges ([IQR], i.e., 25th and 75th percentiles). We also estimated the number of days per week that either a provider or RN was on-site.

We described the percentage of jails employing telemedicine, stratifying by type of care: primary care, mental health, and all other specialties combined. For each care type, we estimated rates of weekly telemedicine per 100 IPW. Similar to on-site personnel coverage rates, we calculated telemedicine coverage rates for “All study jails” and for “Jails using telemedicine” (e.g., only jails with mental health telemedicine were included in our mental health telemedicine rate).

We assessed detention officers’ healthcare responsibilities using 12 items developed based on our earlier qualitative interviews with jail personnel. For each item, we estimated the percent of items answered “Yes.” We also estimated the relationship between total hours of nursing coverage (i.e., total number of hours an RN or LPN was on-site in a week, with a maximum of 168 h), grouped into four categories, and the median number of officer’s healthcare responsibilities. We then tested the relationship between hours of nursing coverage and the number of officer responsibilities using the Wilcoxan-rank sum test.

For all tests of statistical significance, we used a two-sided alpha of 0.05. We conducted analyses using SAS v9.4 (Cary, NC). The University of North Carolina at Chapel Hill Institutional Review Board approved this study.

## Results

### Respondent and Jail Characteristics

Personnel from 73% of county jails in the four states responded. Response rates were 90% in NC, 73% in SC, 63% in GA, and 73% in AL. Among study jails, 33% were located in NC, 13% in SC, 35% in GA, and 19% in AL. Forty-three percent of jails had daily populations of 100 or fewer and 49% of jails were located in rural counties. Ninety-one percent of jails (232/254) contracted with at least one private healthcare entity (Table 1). Surveyed jails accounted for 79% of county jail admissions in the four states, and comparing surveyed and non-surveyed jails, there were no statistically significant differences in the distributions of jail daily population size (*p*=.10) or urbanicity (*p*=.25), but there were differences by state (*p*<.001).

**Table 1:** Jail Characteristics, by Survey Response Status, and Participant Characteristics. A total of 346 Jails Including 254 Responders and 92 Non-Responders

Characteristic	Responders (*n*=254), no. (%)	Non-responders (*n*=92), no. (%)	*P* value^‖^
Jails			
State			
NC	84 (33.1)	9 (9.8)	<0.001
SC	32 (12.6)	12 (13)	
GA	90 (35.4)	53 (57.6)	
AL	48 (18.9)	18 (19.6)	
Daily population^†^			
≤ 50	49 (19.7)	20 (21.7)	0.10
51–100	59 (23.7)	22 (23.9)	
101–200	51 (20.5)	17 (18.5)	
201–500	57 (22.9)	21 (22.8)	
501–1000	23 (9.2)	7 (7.6)	
1001+	10 (4)	5 (5.4)	
County urbanicity^‡^			
Large metro	38 (15.1)	12 (13)	0.25
Medium/small metro	91 (36.3)	26 (28.3)	
Micro/noncore	122 (48.6)	54 (58.7)	
Annual entries^§^	1218577 (79.1)	1540318 (20.9)	
Any contracted healthcare	232 (91.3)	--	
Participant			
Role			
Healthcare supervisor	90 (35.4)	--	
Healthcare provider, no supervision	29 (11.4)	--	
Jail administrator or other non-healthcare personnel	135 (53.2)	--	
Years in position	3.0 (1.2, 8.0)	--	
Gender			
Female	144 (56.7)	--	
Male	107 (42.1)	--	
Missing	3 (1.2)	--	
Race			
White	178 (70.1)	--	
Black	59 (23.2)	--	
Multi-/other	9 (3.5)	--	
Missing	8 (3.1)	--	
Age (years)	48 (40, 56)	–	

^†^5 jails with no population data; population data among non-responders from reference 22

^‡^3 jails with no urbanicity data; urbanicity classification based on reference 23

^§^Row percentages

^‖^*P* values based on chi-square test

In 47% of jails, at least one respondent was a healthcare supervisor or other healthcare personnel; in 53%, the respondent was a jail administrator or other non-healthcare personnel (Table 1). In 9% (23/254) of jails, two people (most often a jail administrator and a healthcare personnel) responded to the survey together. Primary respondents had a median of 3.0 (IQR: 1.2, 8.0) years in their current position. Fifty-seven percent of primary respondents were female and 70% were White with a median age of 48 (IQR: 40, 56) years.

### All and On-site Jail Healthcare Staffing

Among study jails, 96% reportedly had (i.e., on-site, on-call, or via telemedicine) a provider, 93% had nursing staff, 56% had a psychiatric provider, and 72% had a non-prescribing mental health counselor (Table 2). Seventy-one percent of jails had an on-site provider; 90% had on-site nursing including 50% that had RNs and 70% that had LPNs; 9% had on-site psychiatry; and 26% had other mental health personnel on-site.

**Table 2:** Any and On-Site Staffing of Healthcare Personnel in 254 Jails in four US Southeastern States, October 2020–May 2021

Personnel type*	Any healthcare personnel (*n*=254), no. (%)	On-site healthcare personnel (*n*=254), no. (%)	On-site hours All study jails (*n*=254), median (IQR)^¶^	On-site hours “Staffed” study jails, median (IQR)^¶#^
Provider (nonpsychiatric)^†^	243 (95.7)	180 (70.9)	2 (0.0, 4.6)	3.3 (1.7, 6.7)
Nursing	237 (93.3)	229 (90.2)	52.5 (30.5, 78.5)	57 (36.5, 81.7)
RN	170 (66.9)	128 (50.4)	1.1 (0.0, 25.0)	25 (12.0, 52.0)
LPN	205 (80.7)	178 (70.1)	35.5 (0.0, 59.7)	52 (32.1, 72.4)
CNA^‡^	42 (16.5)	--	--	--
Psychiatric providers^§^	141 (55.5)	24 (9.4)	0 (0.0, 0.0)	1.6 (1.0, 3.3)
Mental health (non-prescriber)‖	182 (71.7)	66 (26)	0 (0.0, 1.2)	8.3 (4.3, 13.3)

*RN registered nurse, *LPN* licensed practicing nurses, *CNA* certified nursing assistant, *IQR* interquartile range

^†^Providers consisted of non-psychiatric: Doctor of Medicine (MD), Doctor of Osteopathic Medicine (DO), Physician Assistant (PA), and Nurse Practitioners

^‡^Hours not collected

^§^Psychiatric providers including psychiatric: Doctor of Medicine (MD), Doctor of Osteopathic Medicine (DO), Physician Assistant (PA), and Nurse Practitioners

‖Mental health (non-prescriber) assessed with the item: “Is there a mental health specialist who does NOT prescribe medications—for example, a psychologist, clinical social worker, or counselor--who provides mental health care at your jail?”

^¶^Per 100 Incarcerated person-weeks (IPW)

^#^Personnel-specific estimates based on the population of jails in the column “On-site healthcare personnel (*n*=254), no. (%)”

Among “staffed study jails,” the median time on-site per 100 IPW was 3.3 for providers; 57.0 h for nurses with 25.0 h for RNs and 52.0 h for LPNs; 1.6 h for psychiatrists; and 8.3 h for other mental health personnel. By design, rates of healthcare coverage for all study jails, regardless of on-site personnel, were lower than for the “staffed study jails” (Table 2).

**Figure 1:**
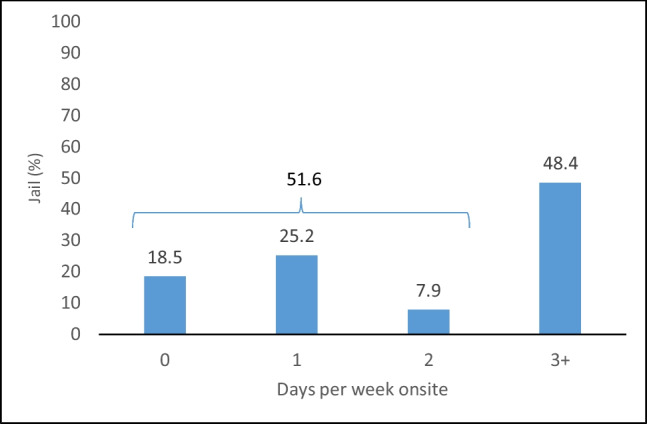
Among 254 jails in the southeast US, the distribution of total number of days in a typical week that either a provider or RN is on site, October 2020–May 2021. In 51.6% of jails, a provider or RN was on site for 2 or fewer days. In 48.4% of jails, a provider or RN was on site for 3 or more days.

Examining the number of days healthcare personnel were on site, we found that in 52% of jails, neither a provider nor an RN was on site for more than 2 days per week (Fig. 1).

### Telemedicine

Telemedicine was reportedly used at least once per month for primary care in 13% of jails, for mental health in 55% of jails, and for other specialty care in 5% of jails. Among jails that reportedly used telemedicine at least monthly for primary, mental health, and other specialty care, respectively, the corresponding median (and IQR) weekly rates of telemedicine (in hours per 100 IPWs) were 2.1 h (0.5, 6.8), 2.1 h (0.5, 4.3), and 0.2 h (0.1, 0.6). Across all study jails, the median telemedicine rates for primary, mental health, and other specialty care were all 0 hours (0, 0–2.2) (Table 3).

**Table 3:** Prevalence and Weekly Rate of Telemedicine use by Healthcare Area Among Jails in Four US Southeastern States, October 2020–May 2021

	Any telemedicine (*n*=254), no. (%)	Telemedicine hours All jails (*n*=254), median (IQR)^*†^	Telemedicine hours among jails using telemedicine, median (IQR)^*†^
Primary care	33 (13)	0 (0,0)	2.1 (0.5, 6.8)
Mental health	140 (55.3)	0.3 (0, 2.2)	2.1 (1.2, 4.3)
Other specialties	12 (4.7)	0 (0,0)	0.2 (0.1, 0.6)

*Interquartile range (IQR); population data missing from 1 jail; telemedicine hours reported per 100 incarcerated person-weeks (IPW) among jails with telemedicine provided at least once per month

^†^Among jails using telemedicine, telemedicine hours were not reported for 5 of 33 jails providing primary care; 6 of 140 jails providing mental healthcare; and 1 of 12 jails providing specialty care. Also, 1 of 140 jails with mental health telemedicine did not have population data. Consequently, telemedicine rates for these jails were not estimated

### Detention Officer Medical Responsibilities

**Figure 2:**
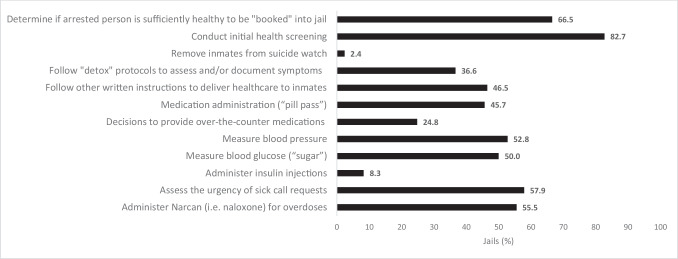
Detention officer healthcare duties in 254 jails in four US southeastern states, October 2020–May 2021.

**Figure 3:**
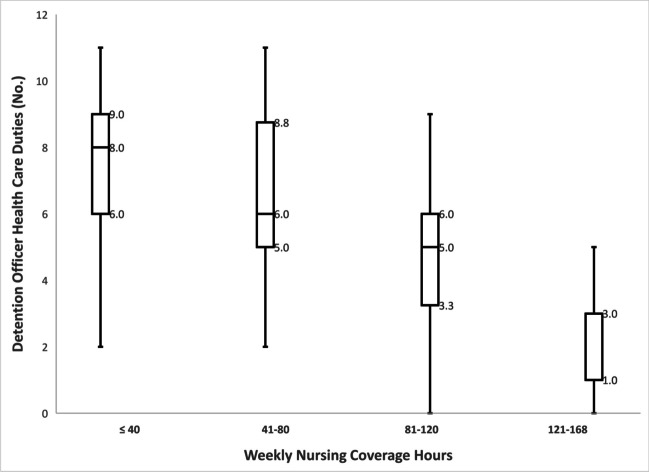
Box and whisker distribution plots of number of detention officer healthcare duties by category of weekly nursing coverage hours in 254 jails in four US southeastern states, October 2020–May 2021*. *Median and interquartile values are labelled; for the Nursing Coverage Category 121–168 h, the 25%tile and median are both 1.0. Based on Wilcoxon Rank Sum Scores, all pairwise comparisons between the four categories of weekly nursing coverage hours were statistically significant (*p* < .05) except for the ≤ 40 vs. 41–80 h (*p* = 0.091).

Detention officers had a wide range of medical responsibilities (Fig. 2). These included deciding at “booking” if an arrested person was healthy enough to be jailed (67%), conducting an intake medical questionnaire (83%), assessing medical request urgency (58%), checking blood glucose (50%), injecting insulin (8%), and following detoxification protocols to assess and document symptom severity (37%) (Fig. 2). The median number of officer responsibilities decreased inversely with greater nursing coverage hours in a week. In jails with 40 or fewer nursing coverage hours, officers had a median of 8 healthcare responsibilities. In jails with 41–80 and 81–120 coverage hours, the median number of responsibilities was 6 and 5, respectively. And in jails with 121 or more coverage hours, the median was 1 (Fig. 3). Across the four categories of nursing hours, pairwise comparisons of the distribution of officer responsibilities were statistically significant except for the comparison between the ≤ 40 and 41–80 h groups (*p* = 0.09).

## Discussion

Despite the tremendous number of people with serious health conditions who are annually jailed, studies have yet to fully capture jail healthcare staffing. We found that the vast majority of jails (96%) reported access to a provider, but providers were seldom on-site. Psychiatric providers’ time on site was particularly low. RNs had a limited on-site presence in most jails, and officers’ healthcare responsibilities were wider in scope in jails with limited or absent nurse staffing.

Our findings of low healthcare staffing corroborate existing albeit limited evidence. In the 2011–2012 nationwide study of jailed people, 39% of respondents with chronic conditions reported not taking their prescription medications because they had not been seen by a doctor. Among all respondents, 57% reported that jail healthcare was “worse” than their care in the prior 12 months and 49% were “not at all satisfied” with care.^3^

While the courts have generally afforded jails latitude in determining appropriate care, the American College of Physicians recently affirmed that jails and prisons should provide “timely access to necessary healthcare services” and that care should be “consistent with that provided to community-based populations.”^24^ Although most jails can use non-routine care, including emergency departments, our findings that neither a provider nor RN was on site for more than 2 days per week in most jails suggest that staffing patterns create a structural barrier to timely care. Further, while head-to-head comparisons with community healthcare settings are imperfect because of different patient case-mixes and lengths of stay, our findings in the context of community regulations and staffing norms suggest that study jails were unlikely to have achieved equivalency.

For example, in community clinics, there are often minimum staffing requirements as in Centers for Medicare and Medicaid Services (CMS)–designated Rural Health Clinics. By regulation, these clinics must have “a physician, NP, PA, certified nurse midwife, clinical social worker, or clinical psychologist on site and available to furnish services.”^25^ Jails’ staffing norms in which providers and RNs are often absent would be deemed unacceptable. Nursing homes provide another comparator that, similar to jails, operate continuously. However, unlike jails, nursing home standards require an RN to be on site 8 daytime hours and LPNs to always be on site.^26^ And in a national survey of residential care facilities—where morbidity is generally less severe than in nursing homes—facilities had greater RN and LPN staffing (averaging 0.2 and 0.17 h per person-day^27^ corresponding to 140 and 120 h per 100 person-week) than in study jails, even when limiting the comparison to “staffed” study jails. Study jails were also less likely to staff RNs (50%) than US public primary and secondary schools (84%).^28^ Beyond community comparisons, psychiatric and non-psychiatric provider hours per person in study jails were less than those of US prisons,^29^ despite jails having a higher disease prevalence.^3^

With providers and RNs infrequently on site, our findings suggest that most jails relied heavily on LPNs and detention officers for healthcare. With 1 year of training, LPNs are skilled in supporting assessments (e.g., collecting vital signs) and following nursing care plans, but not diagnoses, independent nursing assessments, nor creating nursing care plans. Detention officer healthcare training is much less extensive. For example, in NC, the state training program required to become a detention officer includes less than 4 days of health-related instruction. In the absence of on-site RNs and providers, LPNs and officers may be vulnerable to operating without sufficient training and supervision, the lack of which would violate state scope of practice laws;^30–33^ officers can be governed by similar laws when health responsibilities are delegated to them by healthcare personnel. Beyond scope of practice laws, insufficient staffing and supervision can place jails at risk of federal civil rights violations, including violation of the 8th amendment, in the context of demonstrated harms resulting from these staffing patterns.

Finally, the vast majority of jails, 91%, contracted their healthcare to companies or other personnel outside of the jail, suggesting that contracting is much more pervasive than has been recognized previously.^16^

We briefly review a few possible mechanisms to increase jail healthcare staffing. First, the vast majority of jails are financed by their respective counties. An alternative strategy is for the federal and state governments to contribute to jails’ budgets in return for greater oversight. One possible pathway to achieve this change is to end the federal prohibition against using Medicaid/Medicare funds to pay for jail healthcare.^34^ Eliminating this prohibition, particularly in Medicaid expansion states, could enhance jail healthcare funding while requiring that jail healthcare meets CMS accreditation standards.^34^ In a substantial step towards eliminating this prohibition, CMS recently released guidance for states to apply for 1115 Waivers, enabling their use of Medicaid funds to pay for the health services for Medicaid-eligible incarcerated persons up to 90 days prior to release. This type of 1115 demonstration waiver was recently approved for California and is pending in at least 10 other states.^35^ Second, considering our findings of relatively low telemedicine use, another avenue to increase staffing is to enhance routine telemedicine access to RNs and higher level healthcare personnel.^36^ With adequate space and funding, telehealth could reduce jails’ dependency on LPNs and officers to serve as healthcare gatekeepers. Third, most jails contract their healthcare. In developing requests for proposals, jail administrators must first ascertain their populations’ healthcare needs so that they contract for sufficient resources and have mechanisms for oversight.^37^ This oversight should include ensuring appropriate credentialing and the use of metrics to assess clinical performance, the results of which should be available publically. Finally, in response to the COVID-19 pandemic, the custodial population in many jails was temporarily reduced.^38^ Achieving similar reductions by relying more heavily on diversion could help alleviate existing gaps in staffing.

This study has limitations. Participants could have been reluctant to disclose conditions which they deemed reflected negatively on their jails. There may have been recall bias or differential knowledge by participant type (e.g., jail administrators, jail healthcare personnel). Additionally, the survey was developed with cognitive testing and pilot testing, but not full psychometric validation. Finally, our results may not be generalizable to other US regions.

In summary, over the past 50 years, jail healthcare staffing has improved and most jails have nominal access to providers. However, healthcare staffing was typically modest, raising concerns that LPNS and officers may have healthcare responsibilities that exceed their training, and, more broadly, that community healthcare standards likely remain unmet in many jails. Considering the racial and economic disparities in incarceration, these healthcare deficits can further exacerbate health disparities. Given the complexities of jail healthcare and limited county resources, new models of jail healthcare funding, greater telehealth use, and greater accountability of healthcare contractors should be explored as possible mechanisms to adequately address the health needs of jailed persons.

### Supplementary information

ESM 1 (PDF 395 kb)

ESM 2 (PDF 1294 kb)

## References

[1] **Zeng Z.** *Jail Inmates in 2021 - Statistical Tables.* Washington, DC: US Department of Justice, Bureau of Justice Statistics; 2022. NCJ 304888.

[2] Subramanian R, Delany R, Roberts S, Fishman N, McGarry P (2015). *Incarceration's Front Door: The Misuse of Jail in America*.

[3] **Maruschak LM, Berzofsky M, Unangst J.** *Medical Problems of State and Federal Prisoners and Jail Inmates, 2011–12.* Washington, DC: US Department of Justice, Bureau of Justice Statistics; 2015. NCJ 248491.

[4] **Bronson J, Berzofsky M.** *Indicators of mental health problems reported by prisoners and jail inmates, 2011-2012.* Washington, DC: US Department of Justice, Bureau of Justice Statistics; 2017. NCJ 250612.

[5] **Bronson J, Stroop J, Zimmer S, Berzofsky M.** *Drug Use, Dependence, and Abuse Among State Prisoners and Jail Inmates, 2007-2009.* Washington, DC: US Department of Justice, Bureau of Justice Statistics; 2017. NCJ 250546.

[6] Anno BJ (1993). Health care for prisoners. How soon is soon enough?. JAMA..

[7] Modlin HC (1979). Medical care in correctional institutions: the AMA project. Bull Am Acad Psychiatry Law..

[8] American Medical Association (1973). *Medical Care in US Jails: A 1972 Survey*.

[9] *Estelle vs. Gamble,* U.S. 97 429(U.S. Supreme Court 1976).

[10] Schlanger M (2018). The Constitutional Law of Incarceration, Reconfigured. Cornell L Rev..

[11] **Zeng Z, Minton TD**. *Jail Inmates in 2019.* Washington, DC: US Department of Justice, Bureau of Justice Statistics; 2021. NCJ 255608.

[12] **Carson EA.** *Prisoners in 2019.* Washington, DC: US Department of Justice, Bureau of Justice Statistics; 2020. NCJ 255115.

[13] Spaulding AC, Perez SD, Seals RM, Hallman MA, Kavasery R, Weiss PS (2011). Diversity of release patterns for jail detainees: implications for public health interventions. Am J Public Health..

[14] Camplain R, Warren M, Baldwin JA, Camplain C, Fofanov VY, Trotter RT (2019). Epidemiology of Incarceration: Characterizing Jail Incarceration for Public Health Research. Epidemiology..

[15] Potter RH, Lin H, Maze A, Bjoring D (2011). The Health of Jail Inmates: The Role of Jail Population “Flow” in Community Health. Criminal Justice Review..

[16] Jail deaths in America: data and key findings of Dying Inside. Reuters 2020. https://www.reuters.com/investigates/special-report/usa-jails-graphic/. Accessed November 15, 2021.

[17] **Zeng Z, Minton TD.** *Census of Jails, 2005-2019 - Statistical Tables.* Washington, DC: US Department of Justice, Bureau of Justice Statistics; 2021. NCJ 255406.

[18] **Carda-Auten J, Dirosa EA, Grodensky C, et al.** Jail Health Care in the Southeastern United States From Entry to Release. *Milbank Q.* 2022. 10.1111/1468-0009.12569 PMC9576246 35503872

[19] Fenelon A (2013). Geographic Divergence in Mortality in the United States. Popul Dev Rev..

[20] Data are from the Multiple Cause of Death Files, 2018-2021, as compiled from data provided by the 57 vital statistics jurisdictions through the Vital Statistics Cooperative Program. http://wonder.cdc.gov/ucd-icd10.html. Accessed Februrary 2, 2023.

[21] Kaiser Family Foundation. Health Insurance Coverage of the Total Population. 2021; https://www.kff.org/other/state-indicator/total-population/?activeTab=map&currentTimeframe=0&selectedDistributions=employer&sortModel=%7B%22colId%22:%22Location%22,%22sort%22:%22asc%22%7D. Accessed January 20, 2023.

[22] Vera. People in Jail and Prison in Spring 2021. 2021; https://www.vera.org/publications/people-in-jail-and-prison-in-spring-2021. Accessed July 6, 2021.

[23] **Ingram DD, Franco SJ.** NCHS urban–rural classification scheme for counties*. Vital Health. Stat.* 2014; 2(166). 24776070

[24] **Kendig NE, Butkus R, Mathew S, Hilden D,** Health, Public Policy Committee of the American College of P. Health Care During Incarceration: A Policy Position Paper From the American College of Physicians. *Ann Intern Med.* 2022. 10.7326/M22-2370 36410006

[25] Centers for Medicare and Medicaid Services. State Operations Manual Appendix G - Guidance for Surveyors: Rural Health Clinics (RHCs). 2020; https://www.cms.gov/files/document/appendix-g-state-operations-manual. Accessed March 5, 2021.

[26] Center for Medicare Advocay. Nurse Staffing: Requirements of Federal Law. 2014; https://medicareadvocacy.org/nurse-staffing-requirements-of-federal-law/. Accessed January 8, 2022.

[27] **Harris-Kojetin L, Sengupta M, Park-Lee E, et al.** Long-Term Care Providers and Service Users in the United States: Data from the National Study of Long-Term Care Providers, 2013-2014*. Vital Health Statistics.* 2016;3(38). 27023287

[28] Willgerodt MA, Brock DM, Maughan ED (2018). Public School Nursing Practice in the United States. J Sch Nurs..

[29] Pew Charitable Trusts. Prison Health Care: Costs and Qaultity. How and why states strive for high-performing systems. 2017; https://www.pewtrusts.org/~/media/assets/2017/10/sfh_prison_health_care_costs_and_quality_final.pdf. Accessed Sept 23, 2018.

[30] NC Board of Nursing. Decision Tree for Delegation to UAP. 2018; https://www.ncbon.com/vdownloads/position-statements-decision-trees/decision-tree-delegation-to-uap.pdf. Accessed April 23, 2021.

[31] State of Georgia. Standards of Practice for Registered Professional Nurses. In. *Chapter 410-10 STANDARDS OF PRACTICE AND UNPROFESSIONAL CONDUCT.* Vol Rule 410-10-.01

[32] South Carolina Code of Laws Unannotated. In. *Title 40 - Professions and Occupations. Chapter 33, Nurses: Article 1 Nurse Practice Act.*

[33] Alabama Administrative Code. Practice Of Practical Nursing (Licensed Practical Nurse Practice) In. *Section 610-X-6-.05*

[34] Olson MG, Khatri UG, Winkelman TNA (2020). Aligning Correctional Health Standards With Medicaid-Covered Benefits. JAMA Health Forum..

[35] **Mann C, Serafi K, Morgan VE.** States Push for Innovative Ways to Improve Health Outcomes for Justice-Involved Individuals. *To the Point* 2022; https://www.commonwealthfund.org/blog/2022/states-push-innovative-ways-improve-health-outcomes-justice-involved-individuals. Accessed March 23, 2023.

[36] Krsak M, Montague BT, Trowbridge P, Johnson SC, Binswanger IA (2020). Opioid Use and Chronic Infections: The Value of Addressing the Syndemic in Correctional Settings Via Telemedicine Guidance and Broader Use of Long-Acting Medications. J Infect Dis..

[37] Pew Charitable Trusts. Jails: Inadvertent Health Care Providers. 2018; 1-37. Available at: https://www.pewtrusts.org/en/research-and-analysis/reports/2018/01/jails-inadvertent-health-care-providers. Accessed November 15, 2021.

[38] National Academy of Sciences, Engineering, and Medicine (2020). *Decarcerating Correctional Facilities during COVID-19: Advancing Health, Equity, and Safety*.

