# Supplementary material for: Jail Healthcare Staffing in the US Southeast: a Cross-Sectional Survey
Source: J Gen Intern Med. 2023 Oct 26;39(4):603–10. doi: 10.1007/s11606-023-08454-3 (PMC10973318; doi:10.1007/s11606-023-08454-3)
Supplement: Supplementary file 1 — (PDF 395 kb) [file 11606_2023_8454_MOESM1_ESM.pdf]

Appendix A. Survey Development

Survey development was informed by a literature review, qualitative findings from interviews conducted with personnel at 34 jails, and expert input which included study team members with expertise in jail healthcare, emergency medicine, nursing, survey development, and biostatistics**.** After drafting each section, study team members reviewed sections for content, readability, and flow. The study team then conducted cognitive interviews of each section with 5-8 jail healthcare personnel and refined the survey items based on their responses. In total, cognitive interviews were conducted with personnel from 18 jails representing a diversity of facilities based on size, urbanicity, location, and healthcare infrastructure. This iterative process in developing and testing survey sections resulted in the final survey version, which was pilot tested with healthcare personnel in 5 jails. Administration of the pilot survey was deemed successful, and responses from the pilot were included in our final sample.
