# Supplementary material for: Jail Healthcare Staffing in the US Southeast: a Cross-Sectional Survey
Source: J Gen Intern Med. 2023 Oct 26;39(4):603–10. doi: 10.1007/s11606-023-08454-3 (PMC10973318; doi:10.1007/s11606-023-08454-3)
Supplement: Supplementary file 2 — (PDF 1294 kb) [file 11606_2023_8454_MOESM2_ESM.pdf]

Southern Jail
Healthcare Survey

Survey Confirmation

Q1. This is to confirm that you are now ready to administer the Southern Jail Healthcare Survey for **${e://Field/JailName}** in **${e://Field/State}**.

Informed Consent

Q2.
Informed Consent

Purpose
You are being asked to participate in a research study. The purpose of this study is to better understand the internal and community healthcare resources available to jails in the US South. To do so, we are surveying jail personnel about healthcare staffing, the health intake screening process, and jails' use of community-based healthcare resources, such as the emergency department. Ultimately, we hope to understand what's working in jails, find out what healthcare resources jails might need, and to identify ways to reduce costs.

Study Procedures
Participating in the research study is completely voluntary. If you choose to take the survey, you can skip any question you do not wish to answer, and you can end the survey at any time. Deciding not to participate in the survey or stopping the survey before it is finished will not result in any penalties or loss of benefits to which you are otherwise entitled.

The survey will be given by phone. We encourage participants to find a quiet place that is private.
The survey will take around 45 minutes. You will receive a $35 electronic gift card for being a part of this study. We are reaching out to all jails in a 5 state region, and expect that several hundred jail personnel will take the survey.

Benefits and Risks

For all research projects, including surveys, our university asks that we describe any benefits and risks of participating.

The possible benefits to you for taking part in this research are:

While we anticipate that the knowledge from this survey will be informative to jail healthcare personnel, you should not expect to receive any individual benefit from this research study.

The possible risks to you in taking part in this research are:

There are no physical risks. A possible risk is that you may feel uncomfortable answering some of the survey questions, although we have tried to minimize this risk. Again, you not have to answer any questions you do not want to. We also emphasize that you will not be questioned about any sensitive personal information.

How will your information be protected?

To protect your identity as a research subject, survey responses and study records will be stored on a secure server at the University of North Carolina Cecil G. Sheps Center for Health Services Research. All study participants and identifying information will remain confidential.

An important part of conducting research is sharing the results, often through presentations or written articles. All findings from the survey will be reported in summary form, and neither you nor the name of your jail, county, or healthcare company will be identified in any presentations or publications that result from this work.

Certificate of Confidentiality

To help us protect your privacy, we have obtained a Certificate of Confidentiality from our funder, the National Institutes of Health. With this Certificate, the researchers cannot be forced to disclose information that may identify you in any federal, state, or local civil, criminal, administrative, legislative, or other proceedings. You are still able to release information about yourself or your involvement in this research, should you choose to. We do not intend to make voluntary disclosures.

Questions

If you have any questions about this research, please contact the Principal Investigator by calling (919) 962-0752 or emailing david_rosen@med.unc.edu. If you have questions or concerns about your rights as a research subject, you may contact the UNC Institutional Review Board at 919-966-3113 or by email to IRB_subjects@unc.edu.

Q3. **Do you agree to participate in this research study?**

Yes
No

Q4. **Thank you, again, for your willingness to participate in this research by taking our survey. We know you are busy and value your time.**

Again, the purpose of this study is to understand the health resources available to jails. We look forward to learning from your expertise and experience, and hope that you feel comfortable answering candidly.

I’ll ask a question, let you respond, and then may follow up with additional clarifying questions. In some cases, I’ll provide you with response options. If you don’t know the answer to a question, you don't want to answer a question, or if you need to take a break, just let me know.

Before we begin, do you have any questions for me?

Q5. **SECTION 1. PARTICIPANT AND JAIL**

Q6.
Before we begin, can you please confirm the name of your jail facility?

Q7.
Does this jail include multiple facilities? *(Check ONE)*

Yes
No
Don't know

Prefer not to respond
Not applicable

Q8.
For the purpose of this survey, I'm interested in the primary jail facility and those that staff this facility. Would it be possible to respond to my questions today in reference to your primary facility only? *(Check ONE. If jail medical personnel staff multiple facilities, response to this question is No and notes should be added to clarify.)*

Yes
No
Don't know
Prefer not to respond
Not applicable

Q9. **What is the name of your primary jail facility?**

Q10. *OPTIONAL notes to clarify responses (including how/why it is not possible to reference only primary facility in responses):*

Section 1a: Participant Info

Q11. **Now, is it just you in the room taking this survey, or do you have anyone else there with you to help?** *(Mark number indicating TOTAL number of participants in the room)*

1
2

Other:

Q12.
Next, I'd like to ask you a few questions about you and the jail.

What is your job title?

Q13. *(For interviewer only: Check ONE option to categorize based on response to previous question. If needed, probe to determine if participant supervises other staff.*)

Jail administrator

Healthcare leadership role involving the SUPERVISION of other healthcare staff

Healthcare provider that does NOT SUPERVISE other staff

Other:

Q14.
How long have you been working in your current position at this facility? *(Write number of years and/or months in spaces provided, if participant provides this level of detail without probing. For example, if participant says 6 and a half years, write 6 years and 6 months. If participant has worked in facility for less than 1 month, write number of days.)*

Years:

Months:

Days (if less than 1 month):

Don't know

Prefer not to respond

Not applicable

Q15. **Now I am going to ask the same questions of the second participant.**

What is your job title?

Q16.

(For interviewer only: Check ONE option to categorize based on response to previous question. If needed, probe to determine if participant supervises other staff.)

Jail administrator

Healthcare leadership role involving the SUPERVISION of other healthcare staff

Healthcare provider that does NOT SUPERVISE other staff

Other:

Q17.

How long have you been working in your current position at this facility? (*Write number of years and/or months in spaces provided, if participant provides this level of detail without probing. For example, if participant says 6 and a half years, write 6 years and 6 months. If participant has worked in facility for less than 1 month, write number of days.*)

Years:

Months:

Days (if less than 1 month):

Don't know

Prefer not to respond

Not applicable

Section 1b: Jail Info

Q18.

What is your jail's capacity?

Q19. **How many individuals are currently incarcerated in your jail?**

Q20. *If capacity or total population numbers include multiple facilities, please note that here:*

Q21.

Over the past 30 days, how often would you describe your jail as being over-capacity? *(Read response options to participant and check ONE.)*

Always

Very often

Sometimes

Rarely

Never

Don't know

Prefer not to respond

Not applicable

Section 2a: Healthcare Staffing

Q22.
Do you have any medical staff that provide care for INMATES ON-SITE? This could be in-person, on-call, or via telemedicine, and could include mental healthcare. *(Mark no if medical personnel never provides care directly to patients in-person or via telemedicine, and is never on-call)*

Yes

No

Don't know

Prefer not to respond

Not applicable

Q23.
Is there a doctor—either an MD or DO—that provides healthcare at your jail? As a reminder, this could be in-person, on-call, or via telemedicine. *(Only include psychiatrists in response to this question if they provide primary care at the jail.)*

Yes

No

Don't know

Prefer not to respond

Not applicable

Q24. **How many doctors provide healthcare at your jail?**

(If participant provides information about full-time, part-time, PRN, etc., can note that here. If not, do not probe for this--just document total number.)

(Verify that this number does NOT include psychiatrists, unless they also provide primary care at the jail. Optional script for doing this: **And, just to confirm, does this number include psychiatrists?** *If yes*: **Do they also provide primary care at the jail?**)

Q25. **Who do they work for?** *(Check ALL that apply, probe and include notes as needed to categorize*):

Jail/other county entity (excluding local health dept)

Private entity. Please specify if correctional healthcare company:

Local health department

Hospital

Other type of entity:

Don't know

Prefer not to respond

Not applicable

Notes to categorize:

Q26.

Is there a Physician Assistant or Nurse Practitioner that provides healthcare at your jail? This could be in-person, on-call, or via telemedicine. *(Only include psychiatric PAs/NPs in response to this question if they provide primary care at the jail.)*

Yes

No

Don't know

Prefer not to respond

Not applicable

Q27. **How many physician assistants (PAs) or nurse practitioners (NPs) provide healthcare at your jail?**

(If participant provides information about full-time, part-time, PRN, etc., can note that here. If not, do not probe for this--just document total number.)

(Verify that this number does NOT include psychiatric PAs/NPs, unless they also provide primary care at the jail. Optional script for doing this: **And, just to confirm, does this number include psychiatric PAs or NPs?** *If yes*: **Do they also provide primary care at the jail?**)

Q28.

Who do they work for? *(Check ALL that apply, probe and include notes as needed to categorize*):

Jail/other county entity (excluding local health dept)

Private entity. Please specify if correctional healthcare company:

Local health department

Hospital

Other type of entity:

Don't know

Prefer not to respond

Not applicable

Notes to categorize:

Q29.

If only ONE: **Is the [doctor/PA/NP] scheduled to be working ON-SITE at the jail each week?** *(Check ONE)*

If MULTIPLE: **Is at least one [doctor/PA/NP]**) **scheduled to be working ON-SITE at the jail each week?** *(Check ONE. If only come to parking lot to sign charts, mark "no".)*

Yes

No

Don't know

Prefer not to respond

Not applicable

Q30.

How often are they scheduled to be working on-site, if at all? *(Check ALL that apply)*

Onsite according to a regular schedule but less than once a week (e.g. once every two weeks).
Description of when on-site:

Only on-site <u>as needed</u>, rather than on a regular schedule. *Description of when on-site:*

Only come to parking lot to sign patient charts

Never on-site (i.e. care only provided via telemedicine or on-call)

Other. Please specify:

Don't know

Prefer not to respond

Not applicable

Q31.

If ONE: **<u>In a typical week</u>, when is the [doctor/PA/NP] scheduled to be working ON-SITE at the jail?**

If multiple: **<u>In a typical week, when is at least one</u> doctor, PA, or NP scheduled to be working ON-SITE at the jail?**

(Complete table below with information provided by participant, probing as needed. Confirm completeness of data with participant before moving to next question.)

Interviewer tips:

- *If providers SHARE A SCHEDULE, complete first row ONLY (e.g. Each week, either the MD or the PA are on-site M-F 8am-12pm. They alternate every other week, so only one is on-site at a time).*
- *If providers do NOT SHARE A SCHEDULE, complete rows 2 and 3 ONLY.*
- *Include psychiatrists and psychiatric PAs or NPs ONLY if and when providing primary care.*
- *Always specify AM or PM.*
- *If multiple periods within a day, separate with a semi-colon (e.g. 8-10am; 2-4pm)*
- *If 24/7, note using "x" in box of column labeled, "24/7."*
- *If schedule varies, probe for typical or what occurred last week, and document using notes.*

<u>Hours worked onsite by medical providers (MD/DO/PA/NP) each day/night from 12am-12am:</u>

	24/7	Monday	Tuesday	Wednesday	Thursday	Friday	S[illegible]
**1. If providers SHARE A SCHEDULE: Doctors, PAs, and NPs hours combined**							
**2. Doctors**							
**3. PAs/NPs**							

Q32. *Interviewer only: Did you check a box in the table above, indicating that a provider is on-site 24/7?*

Yes

No

Q33. *Optional notes to clarify (only needed if something is out of the ordinary, but no need to add additional details if table provides coverage information):*

Q34.

Is a doctor, PA, or NP ever on-call? *(If needed, read definition: By "on-call," I mean that a [doctor/PA/NP] is available by phone to provide medical guidance or may come into the jail on an as-needed basis to provide patient care.)*

Yes

No

Don't know

Prefer not to respond

Not applicable

Q35. *If jail has only one provider:* **When is the [doctor/PA/NP] on call for the jail?**
If jail has multiple providers: **When is at least one doctor, PA, or NP on call for the jail?**

(EITHER check first option or ALL that apply)

All the time (24 hours a day, 7 days a week)
Evenings and weekends
When a doctor/PA/NP is not on-site at the jail
When a nurse is not on-site at the jail
Other. Please specify:

Don't know
Prefer not to respond
Not applicable

Q36. *Optional notes to clarify responses (IF ON-CALL UNOFFICIALLY, NOTE THAT HERE):*

Q37.
I'm interested in the use of telemedicine at your jail. By "telemedicine," I mean remotely providing healthcare TO PATIENTS using video conferencing or by phone. This does NOT include consultations between providers only. Is telemedicine used to provide care to patients at your jail? *(Can include telemedicine for mental health care. Check ONE)*

Yes
No
Don't know
Prefer not to respond

Not applicable

Q38.
Does your jail use telemedicine to provide primary care, mental health care, or specialty care? *(Check ALL that apply)*

Primary care

Mental health care

Specialty care (other than mental health care). Please specify:

Don't know

Prefer not to respond

Not applicable

Q39. **In a typical week, roughly how many hours is care provided using telemedicine for:**

(Write number of hours per week in boxes provided for each type of care. If *__less frequently than once a week__*, *note hours per month. If* *__less frequently than once per month__*, *indicate this by marking X in box for that column. If don't know, prefer not to respond, or not applicable, indicate this by marking X in box for that column):*

	Hours per week	Hours per month	Less frequently than once per month	Don't know/Prefer not to respond/Not applicable
**Primary care?**				
**Mental health care?**				
**Specialty care (other than mental health care)?**				

Q40.
Some jails have told us about a system called "Arista MD" that allows them to consult electronically with specialists. Does your jail use this or something similar? *(Check ONE)*

Yes
No
Don't know
Prefer not to respond
Not applicable

Q41. *Optional notes on Arista MD use:*

Q42.
Is there a registered nurse, RN, that provides care at your jail? As a reminder, this could be in-person, on-call, or via telemedicine. *(Check ONE)*

Yes
No
Don't know
Prefer not to respond
Not applicable

Q43. **How many RNs provide care at your jail? As a reminder, this includes those that provide care in-person or via telemedicine, and those on-call.**

(If participant provides information about full-time, part-time, PRN, etc., can note that here. If not, do not probe for this--just document total number.)

Q44. **Who do they work for?** *(Check ALL that apply, probe and include notes as needed to categorize*)

Jail/other county entity (excluding local health dept)

Private entity. Please specify if correctional healthcare company:

Local health department

Hospital

Other type of entity:

Don't know

Prefer not to respond

Not applicable

Notes to categorize:

Q45.

Is there a licensed practical nurse, LPN, that provides care at your jail? *(Check ONE)*

Yes

No

Don't know

Prefer not to respond

Not applicable

Q46. **How many LPNs provide care at your jail? As a reminder, this includes those that provide care in-person or via telemedicine, and those on-call.**

(If participant provides information about full-time, part-time, PRN, etc., can note that here. If not, do not probe for this--just document total number.)

Q47. **Who do they work for?** *(Check ALL that apply, probe and include notes as needed to categorize)*

Jail/other county entity (excluding local health dept)

Private entity *(please specify if a correctional healthcare company):*

Local health department

University

Other type of entity:

Don't know

Prefer not to respond

Not applicable

Notes to categorize:

Q48.

Is at least one RN or LPN scheduled to be working ON-SITE at the jail EACH WEEK? *(Check ONE)*

Yes

No

Don't know

Prefer not to respond

Not applicable

Q49.

How often are they scheduled to be working ON-SITE, if at all? *(Check ALL that apply)*

Onsite according to a regular schedule but less than once a week (e.g. once every two weeks). Description of when on-site:

Only on-site as needed, rather than on a regular schedule. Description of when on-site:

Never on-site (i.e. care only provided via telemedicine or on-call)

Other. Please specify:

Don't know

Prefer not to respond

Not applicable

Q50. *If jail has one nurse:* **In a typical week, when is the [RN/LPN] scheduled to working ON-SITE at the jail?**

If jail has multiple nurses: **In a typical week, when is at least one RN scheduled to be working ON-SITE at the jail? When is at least one LPN scheduled to be ON-SITE?**

(Complete table below with information provided by participant. Probe for additional details, if needed to complete table. Confirm completeness of data with participant before moving to next question.)

Interviewer tips:

- *Always specify AM or PM.*
- *If multiple periods within a day, separate with a semi-colon (e.g. 8-10am; 2-4pm)*
- *If 24/7 coverage, note using “x” in box of column labeled, “24/7.”*
- *If schedule varies, probe for typical or what occurred last week.*
- *If RNs and LPNs are interchangeable and share hours, document using third row ONLY.*

Hours worked onsite by each type of nurse each day/night (12am-12am):

	24/7	Monday	Tuesday	Wednesday	Thursday
**1. RNs**					
**2. LPNs**					
**3. RNs/LPNs shared hours**					

Q51. *Interviewer only: Did you check a box in the table above, indicating that a nurse is on-site 24/7?*

Yes

No

Q52. *Optional notes to clarify (only needed if something is out of the ordinary, but no need to add additional details if table provides coverage information):*

Q53.

Is an RN ever on-call? *(If needed, read definition: By "on-call," I mean that an RN is available by phone to provide medical guidance or may come into the jail on an as-needed basis to provide patient care. Check ONE.)*

Yes

No

Don't know

Prefer not to respond

Not applicable

Q54. **When is at least one RN on-call for the jail?** *(EITHER check the first option or ALL that apply):*

All the time (24 hours a day, 7 days a week)

Evenings and weekends

When a doctor/PA/NP are not on-site at the jail

When a nurse is not on-site at the jail

Other. Please specify:

Don't know

Prefer not to respond

Not applicable

Q55.

Optional notes to clarify responses (IF ON-CALL UNOFFICIALLY, NOTE THAT HERE):

Q56.

Is an LPN ever on-call? *(If needed, read definition: By "on-call," I mean that an LPN is available by phone to provide medical guidance or may come into the jail on an as-needed basis to provide patient care. Check ONE.)*

Yes

No

Don't know

Prefer not to respond

Not applicable

Q57. **When is at least one LPN on-call for the jail?** *(EITHER check the first option or ALL that apply):*

All the time (24 hours a day, 7 days a week)

Evenings and weekends

When a doctor/PA/NP are not on-site at the jail

When a nurse is not on-site at the jail

Other. Please specify:

Don't know

Prefer not to respond

Not applicable

Q58.

Optional notes to clarify responses (IF ON-CALL UNOFFICIALLY, NOTE THAT HERE):

Q59.

Is there a certified nursing assistant (CNA), certified medical assistant (CMA), or medical technician (med tech) that provides care at your jail?

Yes
No
Don't know
Prefer not to respond
Not applicable

Q60. **How many CNAs, CMAs, or medical technicians provide care at your jail?**

(If participant provides information about full-time, part-time, PRN, etc., can note that here. If not, do not probe for this--just document total number.)

Q61. **Who do they work for?** *(Check ALL that apply, probe and include notes as needed to categorize)*

Jail/other county entity (excluding local health dept)
Private entity. Please specify if correctional healthcare company:

Local health department
Hospital
Other type of entity:

Don't know
Prefer not to respond

Not applicable

Notes to categorize:

Q62.

Is there a dentist that provides care ON-SITE at your jail*? (Check ONE)*

Yes

No

Don't know

Prefer not to respond

Not applicable

Q63.

How many dentists provide care at your jail?

(If participant provides information about full-time, part-time, PRN, etc., can note that here. If not, do not probe for this--just document total number.)

Q64. **Who do they work for?** *(Check ALL that apply, probe and include notes as needed to categorize):*

Jail/other county entity (excluding local health dept)

Private entity. Please specify if correctional healthcare company:

Local health department

Hospital

Other type of entity:

Don’t know

Prefer not to respond

Not applicable

Notes to categorize:

Q65. **How many hours per week do you have a dentist ON-SITE?** *(If less frequent than weekly, specify hours per month and make note of this. If schedule varies, probe for typical or what occurred last week and note this.)*

Q66.

Not including those that come with EMS in response to calls, is there a paramedic on-site that provides care at your jail?

Yes

No

Don't know

Prefer not to respond

Not applicable

Q67. **How many paramedics provide care at your jail?**

(If participant provides information about full-time, part-time, PRN, etc., can note that here. If not, do not probe for this--just document total number.)

Q68. **Who do the paramedics work for?** *(Check ALL that apply, probe and include notes as needed to categorize)*:

Jail/other county entity (excluding local health dept)

Private entity. Please specify if correctional healthcare company:

Local health department

Hospital

Other type of entity:

Don't know

Prefer not to respond

Not applicable

Notes to categorize:

Q69. **In a typical week, when is at least one paramedic scheduled to be working ON-SITE at the jail?** *(Complete table below with information provided by participant. Probe for additional details, if needed to complete table.)*

Interviewer tips:

- *Always specify AM or PM*
- *If multiple periods within a day, separate with a semi-colon*
- *If 24/7, note using "x" in box of column labeled, "24/7"*
- *If schedule varies, probe for typical or what occurred last week.*
- *If paramedic on-site less than once a week, leave table blank and make notes about schedule in space provided for optional notes to clarify*

Hours worked onsite by paramedic each day/night (12am-12am)

	**24/7**	**Monday**	**Tuesday**	**Wednesday**	**Thursd**
**Paramedic on-site**					

Q70. *Optional notes to clarify (if paramedic is on-site less than once a week, please note schedule here and leave table blank):*

Q71.
Is there a Psychiatrist (MD), Psychiatric Physician Assistant (PA), or Psychiatric Nurse Practitioner (NP) that provides mental health care at your jail? As a reminder, this could be in-person, on-call, or via telemedicine. *(Check ONE)*

Yes
No
Don't know
Prefer not to respond
Not applicable

Q72.

How many psychiatrists, psychiatric PAs, and/or psychiatric NPs provide care at your jail? As a reminder, this could be care provided in-person or via telemedicine, or on-call.

(If participant provides information about full-time, part-time, PRN, etc., can note that here. If not, do not probe for this--just document total number.)

Q73. **Who do they work for?** *(Check ALL that apply, probe and include notes as needed to categorize)*:

Jail/other county entity (excluding local health dept)

Private entity. Please specify if correctional healthcare company:

Local health department

Hospital

Other type of entity

Don’t know

Prefer not to respond

Not applicable

Notes to categorize:

Q74. **Is there a Mental health specialist who does NOT prescribe medications—for example, a psychologist, clinical social worker, or counselor--who provides mental health care at your jail? As a reminder, this could be care provided in-person or via telemedicine, or on-call.** *(Check ONE)*

Yes

No

Don't know

Prefer not to respond

Not applicable

Q75.

How many mental health specialists--for example, psychologists, social workers, or counselors--provide mental health care at your jail?

(If participant provides information about full-time, part-time, PRN, etc., can note that here. If not, do not probe for this--just document total number.)

Q76. **Who do they work for?** *(Check ALL that apply, probe, and include notes as needed to categorize)*

Jail/other county entity (excluding local health dept)

Private entity. Please specify if correctional healthcare company:

Local health department

Hospital

Other type of entity (*include community mental health agency here*):

Don’t know

Prefer not to respond

Not applicable

Notes to categorize:

Q77.

Is at least one mental health provider scheduled to be working ON-SITE at the jail each week? This could include psychiatrists, psychologists, social workers, or counselors. *(Include all prescribing providers and mental health specialists. Probe if needed, and check ONE.)*

Yes

No

Don't know

Prefer not to respond

Not applicable

Q78.

How often are they scheduled to be working on-site, if at all? *(Probe as needed and check ALL that apply.)*

Onsite according to a regular schedule but less than once a week (e.g. once every two weeks). Description of when on-site:

Only on-site as needed, rather than on a regular schedule. Description of when on-site:

Never on-site (i.e. care only provided via telemedicine or on-call)

Other. Please specify:

Don't know

Prefer not to respond

Not applicable

Q79. *If one mental health provider at jail:* **In a typical week, when is the [psychiatrist/psychologist/social worker/counselor] scheduled to be working ON-SITE at the jail?** *If multiple mental health providers at jail:* **In a typical week, when is at least one psychiatrist, psychiatric NP or PA scheduled to be working ON-SITE at the jail? When is at least one psychologist, social worker, or counselor scheduled to be working ON-SITE at the jail?** *(Complete table below with information provided by participant, probing as needed)*

Interviewer tips:

- *Always specify AM or PM.*
- *If multiple periods within a day, separate with a semi-colon (e.g. 8-10am; 2-4pm)*
- *If 24/7, note using “x” in box of column labeled, “24/7.”*
- *If schedule varies, probe for typical or what occurred last week and note this.*

Hours worked onsite by each type of mental health provider each day/night (12am-12am)

	24/7	Monday	Tuesday	Wednesday	Thursday	Fr[illegible]
**1. Psychiatrist/Psychiatric NP or PA**						
**2. Psychologist, social worker, or counselor**						

Q80. *Optional notes to clarify:*

Q81.

Is a mental health provider ever on-call? This could be a psychiatrist, psychologist, counselor, or social worker. *(If needed, read definition: By "on-call," I mean that they are available by phone to provide medical guidance or may come into the jail on an as-needed basis to provide patient care.) (Check ONE)*

Yes

No

Don't know

Prefer not to respond

Not applicable

Q82.

When is at least one mental health provider on-call for the jail? *(EITHER check first option or ALL that apply)*

All the time (24 hours a day, 7 days a week)

Evenings and weekends

When a mental health provider is not on-site at the jail

When a nurse is not on-site at the jail

Other. Please specify:

Don't know

Prefer not to respond

Not applicable

Q83.

Optional notes to clarify responses (IF ON-CALL UNOFFICIALLY, NOTE THAT HERE):

Q84.

Are there any other medical personnel that provide patient care at this jail? *(If no, write No in space provided. If yes, write job titles in space provided.)*

Q85.

You mentioned earlier that there are some times when there are no medical staff on-site. How does your jail handle medical concerns during these times? (*Write response below. Limit to 2-3 sentences max):*

Q86.

You indicated that you have no medical staff on-site. How does your jail typically handle inmate medical concerns? *(Write response below. Limit to 2-3 sentences max):*

Q87. **You indicated that you don't know if your jail has medical staff on-site. How does your jail typically handle inmate medical concerns?** *(Write response below. Limit to 2-3 sentences max):*

Q88. **You indicated that you'd prefer not to discuss your jail's on-site medical staff. How does your jail typically handle inmate medical concerns?** *(Write response below. Limit to 2-3 sentences max):*

Section 2b: Detention Officer Support for Healthcare Provision

Q89.

Now I'm going to ask you some questions about detention officers and the ways in which they may support the provision of healthcare in your jail.

Over the past 30 days, how often would you describe your jail as being fully staffed with detention officers? *(Read response options to participant and check ONE option that best applies)*

Always
Very often
Sometimes
Rarely
Never
Don't know
Prefer not to respond
Not applicable

Q90.

I'm interested in the ways that detention officers may support healthcare in your jail. I'm going to make several statements describing healthcare activities. For each one, I'd like to know if detention officers EVER conduct the activity on their own, <u>without healthcare staff providing input on a case-by-case basis</u>. *(Write "X" in box to indicate Yes or No. ONLY write notes in comment box if needed to clarify. If Don't Know, Prefer Not to Respond, or Not Applicable, indicate by marking X in right hand column. If there is no NARCAN/naloxone at the jail, note this in comments box.)*

Do detention officers EVER, on their own:

	Yes	No	Additional Comments	Don't know/Prefer not to respond/Not applicable
**Decide whether medical clearance is needed BEFORE someone can be booked.**				
**Decide to remove inmates from suicide watch**				
**Follow detox protocols** *(e.g. CIWA or COWS)* **to assess and document severity of symptoms**				
**Follow other written instructions to deliver healthcare to inmates**				

	Yes	No	Additional Comments	Don't know/Prefer not to respond/Not applicable
**Conduct daily pill pass** *(i.e. medication administration)*				
**Make the decision to provide over-the-counter medications--for example, Tylenol--to inmates**				
**Check inmates' blood pressure**				
**Check inmates' blood sugar**				
**Administer insulin injections**				
**Assess the urgency of sick call requests and communicate this to medical staff**				
**Have the ability to administer NARCAN** *(i.e. naloxone)* **in response to potential drug overdose**				

Q91.

In the next set of questions, I'm going to ask you about some of the ways that detention officers may influence inmates' healthcare. *(Read response options and check ONE)*

From your perspective, to what extent do shortages in detention officer staffing affect how quickly inmates are able to receive medical care?

Not at all

A little

Somewhat

Very much

Extremely

Don't know

Prefer not to respond

Not applicable

Q92.

From your perspective, to what extent are detention officers on the lookout for medical needs of inmates?

Not at all

A little

Somewhat

Very much

Extremely

Don’t know

Prefer not to respond

Not applicable

Q93.

From your perspective, how much do detention officers care about the wellbeing of the inmates in this jail?

Not at all

A little

Somewhat

Very much

Extremely

Don’t know

Prefer not to respond

Not applicable

Q94.

From your perspective, to what extent are detention officers able to influence how quickly an inmate receives healthcare in the jail?

Not at all

A little

Somewhat

Very much

Extremely

Don’t know

Prefer not to respond

Not applicable

Q95.
From your perspective, how responsive are detention officers to the medical needs of inmates?

Not at all
A little
Somewhat
Very
Extremely
Don’t know
Prefer not to respond
Not applicable

Section 2c: Safety

Q96.
Next, I’m going to ask you a few questions about safety in the jail. *(Read response options and check ONE.)*

From your perspective, how safe do jail medical staff feel? *(Exclude EMS)*

Not at all safe
A little safe
Somewhat safe
Very safe
Extremely safe
Don’t know
Prefer not to respond
Not applicable.

Q97. **From your perspective, how safe do detention officers feel in this jail?**

Not at all safe
A little safe
Somewhat safe

Very safe
Extremely safe
Don’t know
Prefer not to respond
Not applicable

Q98. **From your perspective, how safe do inmates feel in this jail?**

Not at all safe
A little safe
Somewhat safe
Very safe
Extremely safe
Don’t know
Prefer not to respond
Not applicable

Section 3: COVID-19

Q99.
At this point, you’re about half way finished with the survey.

In the next set of questions, I’d like to learn about the types of things your jail may be doing to handle the COVID-19 pandemic. We know that it is a very difficult time for many jails and that resources may vary significantly across counties.

Are inmates ever screened for COVID using either questions or temperature checks? *(Check ONE)*

Yes
No
Don’t know
Prefer not to respond
Not applicable

Q100.

Are they TYPICALLY screened using: *(Read response options and check ALL that apply)*

Both temperature checks and a questionnaire?

Only temperature checks?

Only a questionnaire?

Other screening methods?

Don't know

Prefer not to respond

Not applicable

Q101.

Do you TYPICALLY screen inmates for COVID: *(Read response options and check ALL that apply)*

At intake? In other words, immediately prior to, during, or within 24 hours after booking?

At release? Specify, if needed:

During incarceration?

Don't know

Prefer not to respond

Not applicable

Q102.
When inmates are screened during incarceration, when and why does this typically happen?

Please specify:

Don't know

Prefer not to respond

Not applicable

Q103. **Next I have a few questions about what TYPICALLY happens if an inmate reports having COVID symptoms.**

If an inmate reports having COVID symptoms, are they TYPICALLY quarantined by themselves or with other inmates? *(Check ONE)*

Yes, by themselves.

Yes, with other inmates.

No. The inmate is not typically quarantined.

Don't know

Prefer not to respond

Not applicable

Q104. **If an inmate reports having COVID symptoms, are they TYPICALLY required to wear a mask?** *(Check ONE)*

Yes

No

Other. Please specify:

Don't know

Prefer not to respond

Not applicable

Q105. **If an inmate reports having COVID symptoms, are they TYPICALLY tested for COVID?** *(Check ONE)*

Yes

Only if symptoms persist or worsen

No

Don't know

Prefer not to respond

Not applicable

Q106. **Next, I have a few questions about what TYPICALLY happens if an inmate tests positive for COVID.**

First, have you EVER had an inmate test positive for COVID in this jail? *(Check ONE)*

(Testing may take place on-site or off-site, but while inmate is in custody)

Yes

No. No inmates have tested positive for COVID in this jail (though inmates may be tested).

No. Inmates are NEVER tested for COVID while incarcerated in this jail.

Q107.

If an inmate tests positive for COVID, are they TYPICALLY housed in a location where they can easily be observed by medical staff? *(Check ONE)*

Yes
No
Don't know
Prefer not to respond
Not applicable

Q108.
If an inmate tests positive for COVID, do inmates that have been in contact with the positive inmate TYPICALLY receive routine symptom or temperature checks? *(Check ONE)*

Yes
No
Don't know
Prefer not to respond
Not applicable

Q109.
If an inmate tests positive for COVID, are they TYPICALLY released early, pending nature of their charges? *(Check ONE)*

Yes
No
Don't know
Prefer not to respond
Not applicable

Q110.
Are inmates that have been in contact with the positive inmate TYPICALLY quarantined by themselves or in groups? *(Check ONE)*

Yes, by themselves.
Yes, in groups.
No, neither.

Don't know

Prefer not to respond

Not applicable

Q111.
If an inmate tests positive for COVID, are all other inmates in the facility TYPICALLY tested? *(Check ONE)*

Yes, all inmates in the facility are tested

No. All inmates are not typically tested, but SOME inmates in the facility are tested.

No. Other inmates are not typically tested in response to a positive case.

Don't know

Prefer not to respond

Not applicable

Q112. **Which inmates are tested?** *(Check ALL that apply)*

Inmates that have been in contact with the positive inmate are tested.

Inmates in the same housing unit as the positive inmate are tested.

Other. Please specify:

Don't know

Prefer not to respond

Not applicable

Q113.
If an inmate tests positive for COVID, are all detention or medical staff required to get tested? *(Check ONE)*

Yes

No, only some detention or medical staff are tested.

No, none are typically tested

Other. Please specify:

Don't know

Prefer not to respond

Not applicable

Q114.

Do inmates without symptoms or contact with a positive case TYPICALLY get *tested* for COVID? *(Check ONE)*

Yes

No

Don’t know

Prefer not to respond

Not applicable

Q115.

How often does this occur? *(Check ALL that apply)*

Weekly

Monthly

Other. Please specify:

Don't know

Prefer not to respond

Not applicable

Q116.
Do staff receive any *routine screening* for COVID? *(Check ONE)*

Yes

No

Don't know

Prefer not to respond

Not applicable

Q117. **How often do staff receive routine screening for COVID?** *(Check ALL that apply)*

Daily. Please specify who is screened and why:

Weekly. Please specify who is screened and why:

Monthly. Please specify who is screened and why:

Other. Please specify frequency, who is screened, and why:

Don't know

Prefer not to respond

Not applicable

Q118.

Some jails have implemented strategies to reduce the likelihood of COVID transmission. Which, if any, of the following strategies are used in your jail? *(Read response options and check ALL that apply)*

More frequent routine cleaning

Greater availability of soap or hand sanitizers

Availability of masks to all inmates

Requirement that inmates wear masks when not in cell

Requirement that detention staff wear masks

Grouping with other inmates booked on the same day (this is sometimes called, "cohorting")

Reduced jail population

Limiting transport off-site (for example, to courts or medical visits)

More frequent use of telemedicine to replace off-site medical visits

Other social distancing strategies (for example, staggered mealtime)

Other. Please specify:

None

Don't know

Prefer not to respond

Not applicable

Q119.

Next I'd like to ask you about the number of tests and positive cases at your facility.

In the past 30 days how many inmates have been tested for COVID-19? *(Testing may take place on-site or off-site)*

Q120. **In the past 30 days, how many inmates have tested positive for COVID-19?** *(Testing may take place on-site or off-site)*

Q121. **What is the biggest challenge your jail faces in addressing the COVID-19 pandemic?** *(Write response below. 2-3 sentences max)*

Section 4a: Medical Intake

Q122.
Okay, you've completed about three quarters of the survey, and we have just a couple more brief sections.

Now that we've discussed the ways that you may be screening for COVID, I'd like to transition to learning about your general medical intake process.

After entering the jail and after medical clearance, are inmates typically asked about their health? This could include questions about suicide, drugs and alcohol, medications, or something else, and is often called the "health intake screening" or "medical intake." *(Verify response for accuracy and check ONE)*

Yes

No

Other. Please specify:

Don’t know

Prefer not to respond

Not applicable

Q123.

Are inmates asked about their health at <u>any</u> time during incarceration? *(Check ONE)*

Only if they place a sick call or have an emergency

Other. Please specify:

Don't know

Prefer not to respond

Not applicable

Q124.

When is this TYPICALLY done?

(Check ONE.)

(Verify with participant that response is based on amount of time since booking and that days are measured in 24-hour periods.)

(Enter number of hours if <24 and number of days if ≥24 hours. Probe for what is typical, but if varies, document the possible range--e.g. 2-4 hours or 1-3 days. If range straddles 24 hour mark, check Other and note range--e.g. 2 hrs-3 days.)

During booking

If less than 24 hrs after booking, number of HOURS after booking:

If equal to or more than 24 hrs after booking, number of DAYS after booking:

Other. Please specify:

Don't know

Prefer not to respond

Not applicable

Q125.

Is this typically done by a medical staff person or detention staff? *(Check ONE)*

Medical staff

Detention staff

When medical staff on-site, medical staff conduct medical intake. When medical staff not on-site, detention officers conduct medical intake.

Other. Please specify:

Don't know

Prefer not to respond

Not applicable

Q126.

If an inmate reports a health issue during the health intake screening process with the detention officer, when do they TYPICALLY meet with medical staff, if at all?

(Check ONE.)

(Verify with participant that response is based on amount of time since booking and that days are measured in 24-hour periods.)

(Probe for what is typical. If it is not possible for the participant to identify a typical timeline, check this response option and provide explanation in space provided.

During booking

If less than 24 hrs after booking, number of HOURS after booking:

If equal to or more than 24 hrs after booking, number of DAYS after booking:

It is not possible for participant to identify a typical timeline. Please explain:

Inmate does NOT meet with medical staff unless they place a sick call

Other. Please specify:

Don't know

Prefer not to respond

Not applicable

Q127.

If an inmate does NOT report a health issue during the health intake screening process, when do they typically meet with medical staff, if at all?

(Check ONE.)

(Verify with participant that response is based on amount of time since booking and that days are measured in 24-hour periods.)

(Probe for what is typical. If it is not possible for the participant to identify a typical timeline, check this response option and provide explanation in space provided.

If less than 24 hrs after booking, number of HOURS after booking:

If equal to or more than 24 hrs after booking, number of DAYS after booking:

It is not possible for participant to identify a typical timeline. Please explain:

Inmate does NOT meet with medical staff unless they place a sick call

Other. Please specify:

Don’t know

Prefer not to respond

Not applicable

Section 4b: Early Release

Q128.

We have heard that inmates with high levels of medical need are sometimes released early or transferred to other facilities. *(Write response in space provided. If don't know, prefer not to answer, or not applicable, note in space)*

In the past 30 days, how many times did your jail transfer inmates to another jail or prison because of their medical needs?

Q129. **In the past 30 days, how many times did your jail require medical clearance before booking?**

Q130. **In the past 30 days, how many times did your jail, AFTER booking, release inmates early because of their medical needs? (for example, via unsecured bond)?**

Section 5a: Use of External Resources (excluding ED)

Q131.

We just have about 10 minutes left.

In this final section, I'd like to learn about whether your jail sends inmates to healthcare providers in the community. And for these next few questions, please don't include Emergency Departments.

Does the jail EVER transport inmates to a healthcare provider outside of the jail? This could include mental health providers. (*Optional:* **Again, this doesn't include EDs.)** *(Check ONE)*

Yes

No

Don’t know

Prefer not to respond

Not applicable

Q132.

<u>How often</u> does the jail typically transport inmates to healthcare providers outside of the jail? (*Optional:* **Again, this could include mental health providers but does NOT include EDs.** *Check ONE.)*

Daily

Less than daily but at least once a week

Less than once a week but at least once a month

Less than once a month but at least every few months

Less than every few months but at least every 6 months

Other. Please specify:

Don't know

Prefer to respond

Not applicable

Q133.

What are the top three conditions for which the jail most frequently transports inmates off-site for care, <u>excluding the emergency department</u>? Again, this could include mental health or substance use-related conditions.

Condition 1:

Condition 2:

Condition 3:

Don't know

Prefer not to respond

Not applicable

Q134. **Do you ever transport inmates off-site for mental health care, excluding the emergency department?** *(Check ONE)*

Yes

No

Don’t know

Prefer not to respond

Not applicable

Q135.

Thinking about the mental health providers that care for your inmates off-site, how satisfied are you with their ability to meet inmates’ mental health needs? Again, this doesn’t include EDs *(Read response options and check ONE)*

Very satisfied

Satisfied

Neutral

Dissatisfied

Very dissatisfied

Don't know

Prefer not to respond

Not applicable

Section 5b: Emergency Department Use

Q136.

Now I'm going to ask some questions about how your jail uses the Emergency Department. For the purpose of this survey, I'm going to refer to the Emergency Department as "the ED." And, in the following questions, I'm interested in use of the ED *after* an inmate has been booked into the jail.

In the last 30 days, how many inmates from your jail were sent to the ED while still in custody?

Q137.

What is the approximate distance (in miles) between your jail and the ED? *(primary facility if multiple used; if don't know miles, request name of ED and street and document here)*

Q138.

How are inmates transported to the ED? *(Check ALL that apply)*

EMS/Ambulance

Patrol cars/ vans (*can include jail, police, or sheriff's vehicles*)

Other (specify):

Don't know

Prefer not to respond

Not applicable

Q139. **Which is used <u>most often</u> to transport inmates to the ED?** *(Check ONE)*

EMS/Ambulance

Patrol cars/ vans (can include jail, police, or sheriff's vehicles)

Other (specify):

Don't know

Prefer not to respond

Not applicable

Q140.
How often is transport to the ED delayed due to shortages in detention officer staffing?
(Read response options and check ONE)

Always

Often

Sometimes

Rarely

Never

Don't know

Prefer not to respond

Not applicable

Q141.
Do you ever send inmates to the ED for a medical issue that is NOT an emergency?
(Check ONE)

Yes

No

Don't know

Prefer not to respond

Not applicable

Q142.

What are some reasons that you might do this? *(Allow participant to respond and only check those that are noted without prompting. Clarify responses, as needed, and check ALL that apply)*

No nursing staff on-site when care needed

No providers (MD/NP/PA) on-site when care needed

Insufficient medical equipment on-site to provide care needed

Care needed after hours when other healthcare providers in the community were unavailable

Other. Please specify:

Don't know

Prefer not to respond

Not applicable

Section 5c: Medical Equipment

Q143.

Now I'm going to list some medical equipment. For each piece of equipment, I'd like you to tell me whether it's available in your jail. *(Check ALL that apply. Only include equipment permanently stationed at jail, and not brought on-site as-needed.)*

X-ray machine

Ultrasound machine

Equipment to place sutures

Equipment to remove sutures

Dermabond

EKG machine

None of the above

Don't know

Prefer not to respond

Not applicable

Section 5d: Relationship with ED/EMS

Q144.

In the next section, I'd like to learn more about your jail's relationship with the ED and EMS. Please tell me whether each of the following statements is very true, somewhat true, or not true for your jail. *(Read response options to participant and check ONE. If jail sends inmates to multiple emergency departments, ask them to think about the one they use most frequently.)*

We try to send patients to the ED as little as possible.

Very true

Somewhat true

Not true

Don't know

Prefer not to respond

Not applicable

Q145.

We try to call EMS as little as possible.

Very true

Somewhat true

Not true

Don't know

Prefer not to respond

Not applicable

Section 5e: Emergency Medical Services (EMS)

Q146.

Now I would like to ask some additional questions about EMS.

In the past 30 days, how many times was EMS called to your jail?

Q147. **When EMS comes to your jail, do they EVER assess the inmate but <u>not</u> transport to the ED?** *Check ONE)*

Yes

No

Don't know

Prefer not to respond

Not applicable

Q148.

What percentage of EMS visits would you estimate are assessments without transport?

Q149.

Does EMS bill *(the jail, healthcare company, or other)* **for transports to the ED?** *(If yes, probe to determine whether always or sometimes. Check ONE)*

Yes, always

Sometimes

No, never

Don't know

Prefer not to respond

Not applicable

Q150.

Is your jail billed when EMS comes to assess inmates but does not transport them to the ED? *(Check ONE)*

Yes, always

Sometimes

No, never

Don’t know

Prefer not to respond

Not applicable

Section 6: Closing

Q151.

I have a few final questions before we wrap up today.

What is the biggest challenge your jail faces in providing healthcare to inmates? *(Limit response to 2-3 sentences.)*

Q152.

We’ve heard that caring for inmates with mental illness can be challenging for many jails.

What are some of the biggest challenges that your jail faces in supporting inmates with mental illness? *(Only check those that are noted without prompting. Clarify responses, as needed, and check ALL that apply)*

High volume of inmates with mental illness

Insufficient number of mental health staff at the jail

Inability to provide the medications that inmates need

Limited number of offsite mental health agencies

Offsite mental health agencies are of poor quality

Difficult to access services at offsite mental health agencies

Limited number of mental health hospital beds

None

Other, please specify:

Don't know

Prefer not to respond

Not applicable

Q153. **Is there an innovative practice or something you're proud of doing at your jail?** *(Limit response to 2-3 sentences.)*

Q154.

To close today, I have four demographic questions for you.

What educational degree(s) or certifications have you obtained? *(Check ALL that apply)*

Licensed practical nurse (LPN)

Registered nurse (RN)

Physician Assistant (PA) or Nurse Practitioner (NP)

Medical Doctor (MD) or Doctor of Osteopathic Medicine (DO)

Other, please specify:

Don't know

Prefer not to respond

Not applicable

Q155.

With what race or races do you identify? *(Check ALL that apply)*

American Indian or Alaska Native

Asian

Black or African American

Hispanic or Latino

Native Hawaiian or Other Pacific Islander

White

Don't know

Prefer not to respond

Not applicable

Q156.

How old are you?

Q157.

What is your gender?

Female

Male

Other. Please specify:

Don't know

Prefer not to respond

Not applicable

Q158.

Thank you so much for the time that you've dedicated to this survey today. We know that you're busy and truly appreciate your contributions to this work. The information you've shared today, along with the responses from the other jails in our study, will be summarized and disseminated. We hope that results will allow jails to learn from one another and policymakers to learn from you and your colleagues.

We'd like to be able to share the results of this study with you. Is this something you'd be interested in? If so, what's the best way to get these to you (e.g. mail, email)?

Yes. Preferred contact method and contact information:

No

Other

Don't know

Prefer not to respond

Not applicable

Q159.
Also, as we’re reviewing the information gathered in these surveys, we may have additional clarifying questions. Would it be okay if a study team member contacted you with follow-up questions?

Yes

No

Other, please specify:

Don't know

Prefer not to respond

Not applicable

Q160.
Finally, I’d like to send you a $35 electronic gift card to thank you for your time today. The email address is have on file for you is ________. Is that correct?

Yes

No. Use this email address instead:

Cannot receive gift card

Don't know

Prefer not to respond

Not applicable

Q161. *For interviewer only*: Please write anything notable about this survey here, e.g. participant called a colleague for help answering a question, they got interrupted for a long time, they seemed distracted, seemed to be fabricating responses, etc.

Powered by Qualtrics
